# Retraction: Stress-mediated Sin3B activation leads to negative regulation of subset of p53 target genes

**DOI:** 10.1042/BSR-2015-0122_RET

**Published:** 2024-07-03

**Keywords:** gene regulation and bleomycin-induced stress, genotoxic stress, p53, Sin3-histone deacetylase (HDAC) complex, Sin3B

This article is being retracted from *Bioscience Reports* at the request of the Editor-in-Chief and the Editorial Board following receipt of a notification from a reader, alerting the Editorial Board to multiple similarities between the figures of the paper, specifically between:

- Figure 1A p53 panel and Figure 2C RNF220 panel with horizontal flip transformation
- Figure 5C KB Bleomycin - Sin3B band and Figure 5C A549 Bleomycin + β-actin band
- Figure S2 Saos2 18S rRNA panel and Figure S2 H1299 Sin3B panel

The authors were contacted regarding the concerns raised, and provided replacement data for Figure 1A, Figure 5C, and Figure S2 which support the paper's original conclusions. However, given the extent of the issues identified in the original figures and in line with the journal's Editorial Policy, the Editorial Board stands by the decision to retract. The authors agree with the retraction.

